# Comparative study on the influence of rare earth ion doping on the structural and optical properties of simple B_2_O_3_–Na_2_O glasses

**DOI:** 10.1038/s41598-025-21224-x

**Published:** 2025-10-07

**Authors:** M. M. El-shabaan, Amaal Mohamed, M. I. Youssif, N. A. El-Ghamaz, E. M. Ahmed

**Affiliations:** 1 Physics Department, Faculty of Science, Damietta University, New Damietta, 34517 Egypt; 2 Department of Engineering, College of Engineering and Technology, University of Technology and Applied Sciences, Suhar, 311 Oman

**Keywords:** RE-doped sodium borate glass, XRD, FT-IR, UV–Vis, Refractive index, Glasses, Materials science, Physics

## Abstract

In this study, a series of sodium borate glasses with the general composition 50 mol% B₂O₃ – (50–x) mol% Na₂O – x mol% RE₂O₃ (where RE = La, Nd, Gd, Ho, Er, Yb and x = 0 and 1 mol%) were synthesized via the melt-quenching technique. This study aims to systematically investigate the effect of substituting different rare earth ions into a fixed sodium borate glass matrix to understand their role in modifying structural and optical properties. XRD analysis confirmed the amorphous nature of all glass samples. FTIR spectra showed structural transformations marked by an increase in BO₄ units and suppression of boroxol rings with rare earth doping. Density and molar volume increased from 2.42 to 2.49 g/cm^3^ and from 27.17 to 27.74 cm^3^/mol, respectively, reflecting the influence of RE ionic radii and field strengths on glass compactness. Optical absorption measurements revealed a shift in the absorption edge, with band gap values ranging from 3.13 to 3.38 eV. Tauc, ASF, and HEM models indicated direct allowed transitions in all samples. The refractive index ranged from 2.299 to 2.361. These results demonstrate that rare earth ions act as effective modifiers, enabling tunable structural and optical properties in sodium borate glass systems, which are promising for photonic and radiation shielding applications.

## Introduction

Oxide glasses are versatile materials with diverse applications, ranging from everyday simple items to advanced technologies^1^. Some glasses can be viewed as ionic polymers with network structures composed of oxygen atoms linked through multivalent atoms like boron, silicon, or phosphorus^2^. Their properties are influenced by network parameters such as crosslink density and oxygen atom packing density^2^. Various studies are focusing on transition metal ions and their roles in glass properties^3,4^. Oxide glasses exhibit unique mechanical properties, such as high stiffness and sharp fracture edges, which have made them useful since ancient times^5^.

Borate glasses are technologically important materials with diverse applications in optics and luminescence^6–9^. Their structure is characterized by planar BO_3_ groups and boroxol rings, which can be modified by the addition of metal cations^10^. The fraction of fourfold coordinated boron atoms in these glasses can be predicted using models based on crystalline polymorphs^11^. Borate glasses exhibit unique optical properties, including anomalous partial dispersion, high refractive indices, and nonlinear optical characteristics^12^. These properties make them suitable for various applications, such as optical glasses, white LEDs, and laser sources^13^. The addition of rare earth elements and heavy metals can further enhance their luminescence properties to be more suitable for use in display technologies^12,13^. The structural and optical properties of borate glasses can be tailored by adjusting their composition, making them versatile materials for a wide range of applications.

Adding different amounts of Na_2_O to the pure borate glass is found to enhance the physical properties of such glasses. Q. Xu et al. reported that the increase of Na_2_O content in sodium borate glass rises the fraction of four-coordinated boron atoms, leading to network development up to about 33 mol% Na_2_O, after which the network begins to disintegrate^14^. G.E. Jellison et al. showed that the spectra for sodium borate glasses are sensitive to the different structural groups in the glasses. They reported that, NMR studies have identified five distinct boron sites in these glasses, including two 4-coordinated and three 3-coordinated sites. The two 4-coordinated boron sites were identified as BO_4_ units connected to: (1) all BO_3_ units, and (2) one BO_4_ unit and three BO_3_ units. The three 3-coordinated boron sites were identified as BO_3_ units connected to: (1) all BO_3_ units, (2) a mixture of BO_3_ and BO_4_ units, and (3) all BO_4_ units^15^. S.P. Jaccani et al. concluded that the elastic properties of sodium borate glasses *x*Na_2_O–(100-*x*)B_2_O_3_ (*x* = 10, 15, 20, 25, 30, and 35 mol%) are sensitive to thermal history and composition, with anomalous increases in elastic moduli observed in some air-cooled glasses just below their glass transition temperature^16^.

Recent studies have explored rare earth-doped sodium borate glasses for photonic and radiation shielding applications. For examples: Er_2_O_3_-doped glasses showed emissions at 550, 660, and 1550 nm, with potential for telecom C-band devices due to their 1.5 μm emission^17^. Also, doping of NiO-BaO-B_2_O_3_ glasses with La_2_O_3_ was reported to enhance the radiation shielding properties, and to increase the BO_4_ content and improving glass structure^18^. Furthermore, Gd_2_O_3_-doped borate glasses was found to exhibit an improvement in the radiation shielding and mechanical properties, with the best results observed at 3 mol% Gd_2_O_3_ concentration^19^. A. Alemi et al. showed that, the Nd_2_O_3_-doped In_2_O_3_-Na_2_B_4_O_7_ glasses exhibited a strong ^4^I_9/2_ → ^4^G_5/2_ + ^2^G_7/2_ transition at 584 nm and fluorescence at 800–900 nm^20^. J. T. James et al. reported that, the Nd^3+^-doped Na_2_O-CaO-B_2_O glasses demonstrated high emission cross-sections and quantum efficiencies, making them suitable for 1.05 μm laser applications^6^.

Additionally, several studies have examined the effects of modifying other borate systems. For instance, the impact of TeO₂ variation on thermal properties in lead borate glasses doped with Pr₂O₃ significantly altered thermal stability and glass-forming ability^21^. Sm₂O₃-doped lithium zinc borate glasses showed enhanced photoluminescence and improved structure with increased modifier content^22^. The incorporation of Bi₂O₃ and Sm₂O₃ into phospho-zinc tellurite glasses revealed complex nonlinear optical behavior and structural shifts^23^. Moreover, Sm^3^⁺-activated lithium zinc borate glasses exhibited high nonlinear optical coefficients in both femtosecond and nanosecond regimes, suitable for ultrafast photonics^24^.

In addition to rare-earth doping, numerous studies have investigated the role of transition metal and heavy metal oxides in tuning the functional properties of various glass systems. For instance, Kumar et. al.^25^ studied Sb₂O₃–NiO co-doped lithium fluoro borophosphate glasses and observed enhanced dielectric dispersion and optical response. CoO-doped alkali bismuth phosphosilicate glasses showed significant thermoluminescence and photoluminescence enhancement, making them suitable for sensing and display applications^26^. Likewise, the incorporation of Al^3^⁺ and Sb^3^⁺ in Pr₂O₃- and Nd₂O₃-doped PbO–SiO₂ glasses led to improved infrared emission and 1.06 μm lasing potential, demonstrating the effects of multi-dopant synergy on optical transitions^27,28^. Cu^2^⁺ and Ni^2^⁺ ions were found to induce defect centers and improve dielectric and TL behaviors in various bismuth, silicate, and aluminate glasses^29,30^. Sm₂O₃ and Cu^2^⁺ doping in silicate systems also produced strong thermoluminescence and radiation shielding performance, indicating multifunctional applicability^31,32^. These findings emphasize how targeted doping strategies can be used to engineer glass properties for specific photonic, optoelectronic, and shielding functions—paralleling the goals of the present work, which compares six different RE ions in a unified borate base matrix.

Despite these findings, a comprehensive comparative study on multiple RE ions (e.g., La, Nd, Gd, Ho, Er, Yb) in a unified sodium borate glass matrix remains limited. This work aims to fill that gap by systematically substituting 1 mol% of Na⁺ with 1 mol% of each RE^3^⁺ ion in a 50 mol% B₂O₃ – 50 mol% Na₂O glass to explore changes in structural and optical properties for potential applications.

## Experimental techniques

A series of sodium borate glasses with the general formula 50 mol% B₂O₃ – (50−x) mol% Na₂O – x mol% RE₂O₃, where x is 0 and 1, (RE = La, Nd, Gd, Ho, Er and Yb) were synthesized using the conventional melt-quenching technique. Analytical-grade reagents were used, including boric acid (H₃BO₃, 99.9%, Sigma-Aldrich), sodium carbonate (Na₂CO₃, 99.9%, Sigma-Aldrich), and the respective rare earth oxides (RE₂O₃, ≥99.99%, Sigma-Aldrich). The raw materials were weighed using an electronic balance with ±0.0001 g accuracy, thoroughly mixed in an agate mortar for at least 15 minutes to ensure homogeneity, and then transferred to high-purity porcelain crucibles. Each batch had a total weight of approximately 20 g. Melting was performed in an electric furnace at 1000 °C for 1 hour in air, with intermittent stirring using a platinum rod to improve homogeneity. The molten glass was rapidly quenched by pouring onto a pre-heated stainless steel plate and pressed with another steel plate**.** The quenched samples were immediately annealed at 400 °C for 3 hours to relieve internal stresses and then cooled slowly to room temperature inside the furnace. The resulting glass samples were cut and polished using silicon carbide papers followed by 0.3 μm alumina slurry to obtain optically smooth surfaces. All samples were stored in desiccators to prevent moisture absorption. The amorphous nature of the glasses was examined using X-RAY DIFFRACTION (XRD) D2 PHASER, equipped with the unique LYNXEYE XE-T detector, CuKα and CoKα X-ray sealed tubes (focus size – 0.4 x 12 mm), Rated operation X-ray sources: 30 kV/10 mA (offers an angular accuracy better than ±0.01° 2θ with a step size of 0.02° and a detection limit of ~1% for crystalline phases). Diffraction patterns were recorded automatically for 2θ in the range 0º - 100º with a scanning speed of 0.03º/min. Fourier transform infrared (FT-IR) spectra was measured at room temperature using a 4100 JASCO Michelson interferometer (has a resolution of 1 cm⁻^1^ and wavelength accuracy of ±0.01 cm⁻^1^) with the KBr disk method. Density was measured at room temperature according to the Archimedes technique using a digital density balance (sensitivity: ±0.0001 g/cm^3^), using toluene (density ≈ 0.8635 g/ml) as the immersion liquid. UV–Vis absorbance spectra for polished bulk samples were recorded using a JASCO-V630 double-beam spectrophotometer (has a wavelength accuracy of ±0.3 nm and a photometric repeatability of ±0.002 Abs) over a wavelength range of 200 to 1100 nm. The thickness of glasses is 6.35 mm for B-Na, B-Na-La, B-Na-Nd, B-Na-Gd, and B-Na-Ho samples and 3.8 mm for B-Na-Er and B-Na-Yb samples.

## Results and discussion

### Structural properties

#### Glass appearance, XRD and EDX analyses

**Fig. 1:**
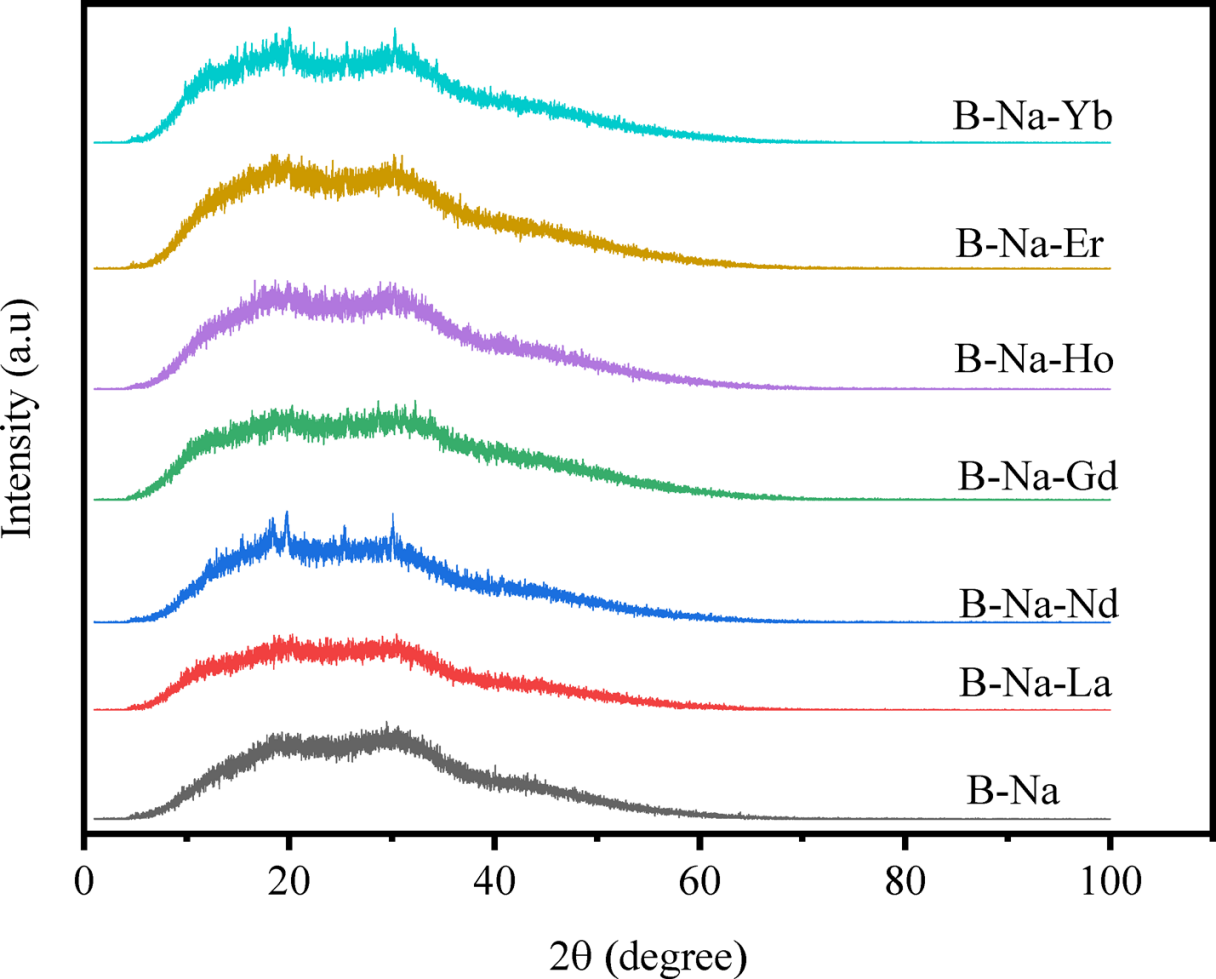
XRD patterns of the B_2_O_3_-Na_2_O-RE_2_O_3_ glasses.

**Fig. 2:**
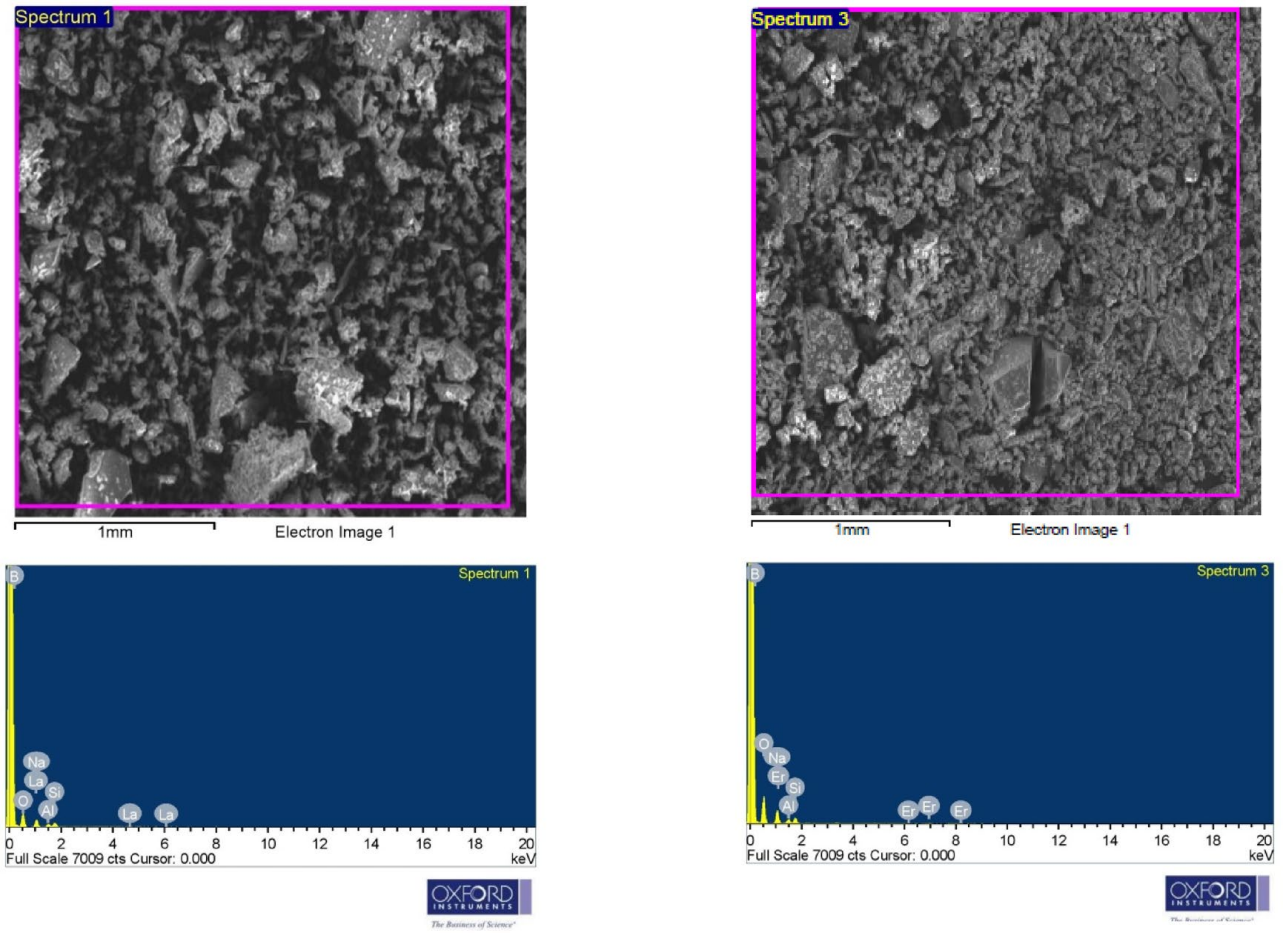
The EDX spectrum for the La_2_O_3_ and Er_2_O_3_ doped B_2_O_3_-Na_2_O glasses.

The X-ray diffraction (XRD) patterns of the glass samples were analyzed to explore their nature, as illustrated in Fig. 1. Each of the displayed curves represent a superposition of two broad humps centered at 2θ ≈ 19° and 30° without any sharp peaks. The absences of discrete or sharp peaks in these charts confirm the high degree of amorphousness of all the prepared glasses. Also, the humps of all the glasses did not show angle shifts with respect to each other, which in turn suggests that the construction of the B-Na base glass doesn’t affected by the introduction, as well as, the replacement of the different RE oxides. Similar results has been reported for sodium borate glass with the composition (70%B_2_O_3_-(30-x)%Na_2_O-x%Y_2_O_3_ with x = 0, 0.5, 1, 1.5, 2, and 2.5 mol% )^33^. The photo images of the as-manufactured glasses are displayed in Table 1. The images reflect the colors, clearness, and transparency of the obtained glasses. The B-Na glass sample appeared colorless, while the rest of the samples showed to present distinct colors. The apparent colors reflect some of the distinct color characteristics of each added RE oxide. Furthermore, the elemental analysis of two random samples was checked out using an Energy-Dispersive X-ray (EDX) spectrometer (Oxford Instruments, U.K.), to explore the chemical compositions of the glasses and their contributions. The spectrum in Fig. 2 represents all the expected elements with their stoichiometric portions, in addition to a very low percentage of the contaminated elements Si and Al, with ratios less than 0.06 Atomic%. The usage of the porcelain crucibles in the melting process is supposed to be the source of observed contamination.

**Table 1:** Sample Code, compositions, and photos of the studied glass samples.

Sample Code	Glass Composition (mol%)								Sample photo
	B_2_O_3_	Na_2_O	La_2_O_3_	Nd_2_O_3_	Gd_2_O_3_	Ho_2_O_3_	Er_2_O_3_	Yb_2_O_3_	
B-Na	50	50	–	–	–	–	–	–	
B-Na-La	50	49	1	–	–	–	–	–	
B-Na-Nd	50	49	–	1	–	–	–	–	
B-Na-Gd	50	49	–	–	1	–	–	–	
B-Na-Ho	50	49	–	–	–	1	–	–	
B-Na-Er	50	49	–	–	–	–	1	–	
B-Na-Yb	50	49	–	–	–	–	–	1

#### FT-IR spectroscopy

**Fig. 3:**
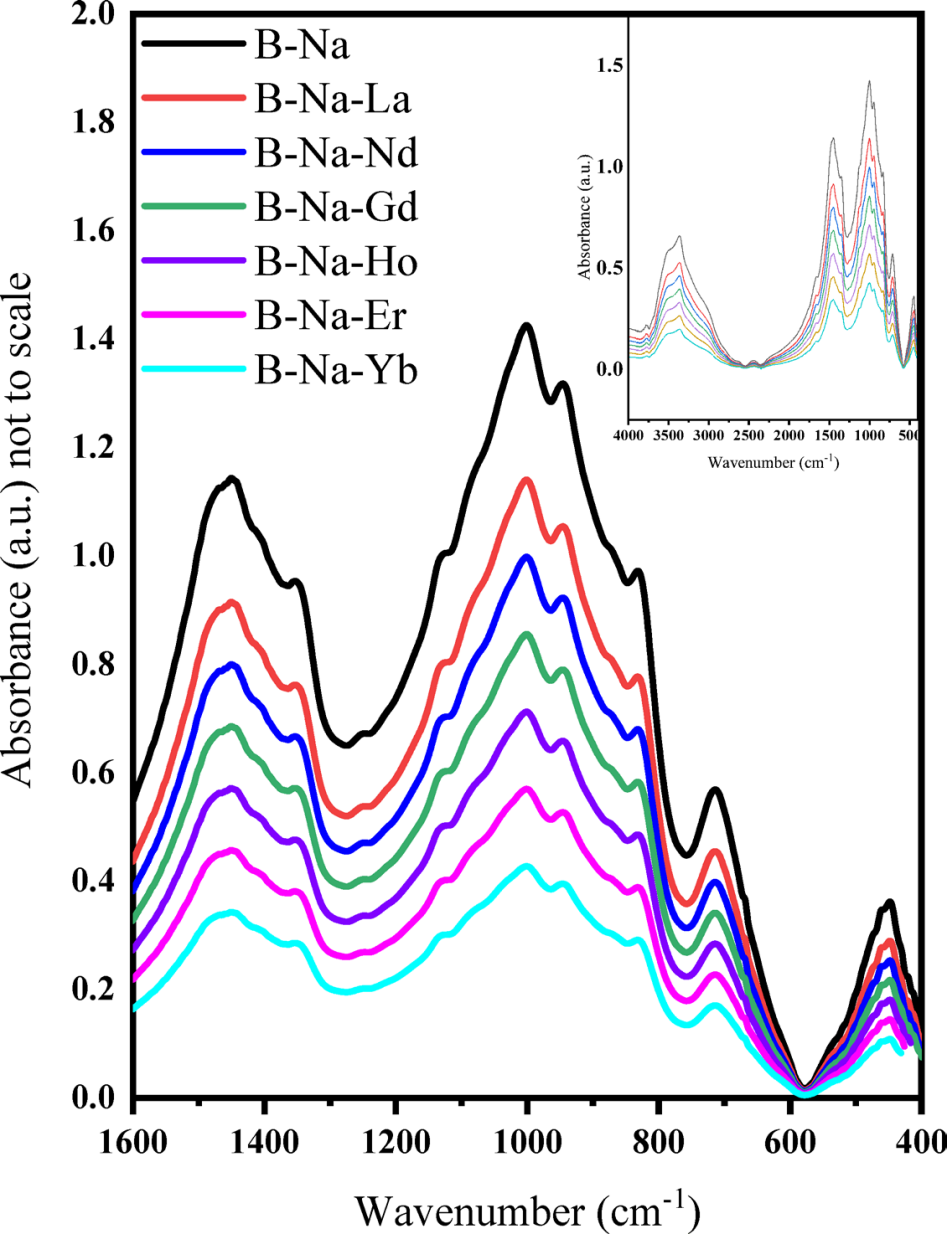
FT-IR spectra of the studied B_2_O_3_-Na_2_O-RE_2_O_3_ glasses.

FT-IR spectroscopy is a vital technique for determining the types of bonds and groups shaped within the network of various materials. The FT-IR spectra of the examined glasses were recorded in the range of 4000 to 400 cm^-1^, as shown in Fig. 3. The presence of water and/or hydroxyl groups is indicated by bands appearing at above 1700 cm^-1^^33^ (see the inset of Fig. 3). The significant FT-IR spectra are observed in the range of 1600–400 cm^-1^, which are chosen for detailed analysis and are zoomed in Fig. 3. A broad band detected between 1240 and 1600 cm^-1^ is attributed to the asymmetric stretching of the B–O bond in trigonal BO_3_ units^34^. This band is centered at ~ 1460 cm^-1^ for the B-Na sample, and shifted to ~ 1410 cm^-1^ for all the RE doped samples. The clear shoulder around 1350 cm^-1^ in the B-Na sample is mainly owing to the B–O stretching vibrations in BO_3_ groups^35^. This shoulder is found to be dissolved completely with the addition of different REs to the glass matrices. The vanishing of this well-known shoulder at 1350 cm^-1^, in B-Na-Er sample may be taken as an evidence of the formation of B–O–Er stretching bond. Fine shoulders at ~ 1190 and ~ 1130 cm^-1^ are recommended for the stretching vibrations of tetrahedral BO_4_ groups^36^. The broad band in the range 1170–780 cm^-1^ is observed for all the glasses. This band may be also resulted from the B–O bond stretching in tetrahedral BO_4_ units^34^. An additional sharp band at about 715 cm^-1^ is related to B–O–B stretching in metaborate rings^34^. Another sharp band at ∼460 cm^-1^ is confirmed to be attributed to the vibrations of Na–O bonds in their network sites^36^.

**Fig. 4:**
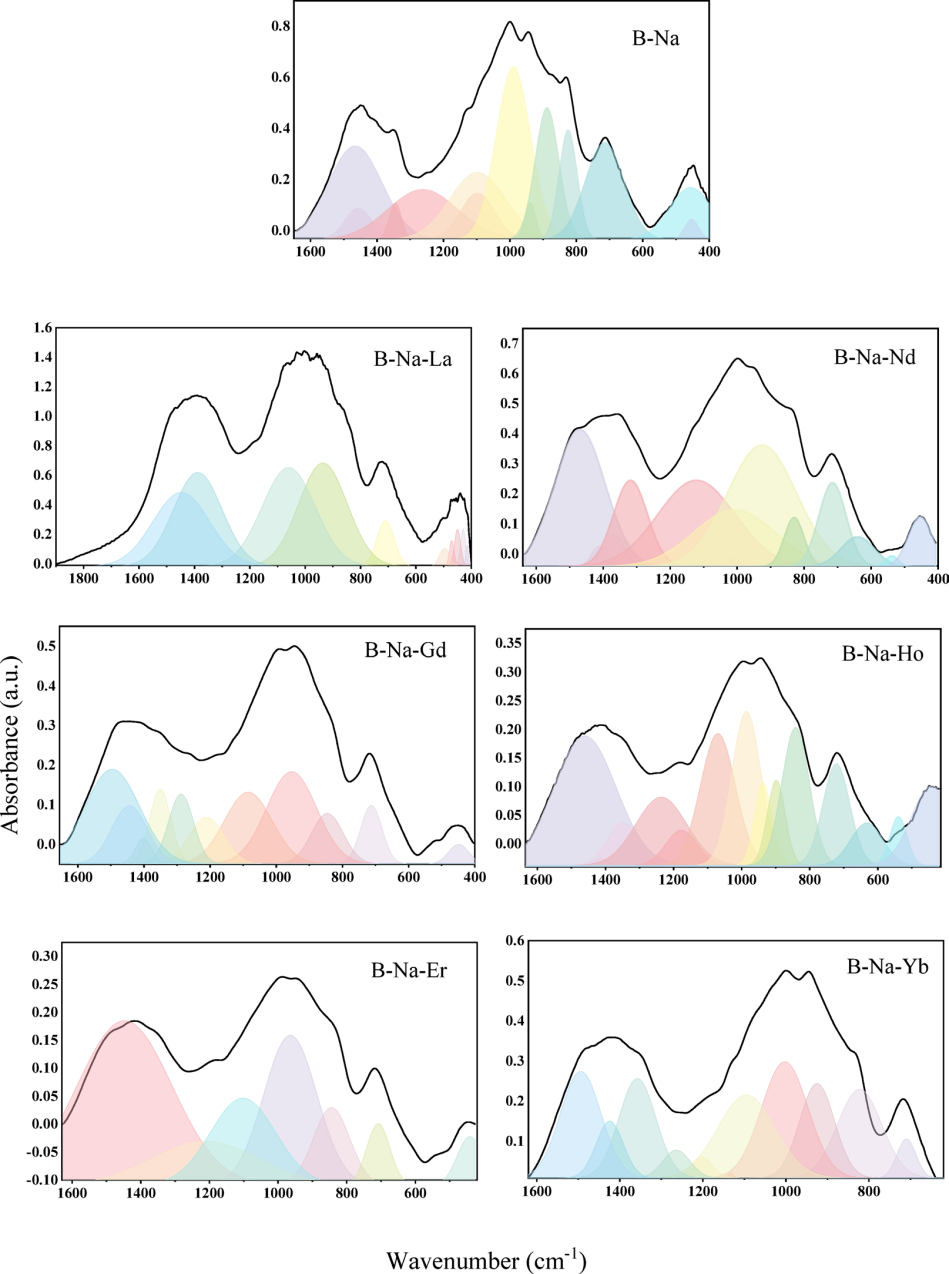
Deconvoluted FT-IR spectra of the B_2_O_3_-Na_2_O-RE_2_O_3_ glass system.

The broad spectra that resulted from the local distribution of molecules in the network was decomposed into deconvoluted peaks using the Gaussian function included in the Originlab™ software (OriginPro 2018). Figure 4 illustrates the deconvoluted peaks of the studied glasses and the deconvoluted data for each peak, including area (A) and center (c) are recorded in Table 2. The range of 1495–1401 cm^-1^ is attributed to anti-symmetric stretching vibrations of triangular (BO_3_)^3−^ with three NBOs from various borate groups^37^. The peak around 1388–1318 cm^-1^ is due to B–O^-^ asymmetrical stretching vibrations in BO_3_ triangular units and/or borate triangles with BO_2_O^-^ and/or NBO units in various borate groups^37^. B–O stretching vibrations in BO_3_ structural units are present in the range of 1287–1202 cm^-1^^38^. In the range of 1178–1102 cm^-1^, asymmetrical stretching of the B–O bond in trigonal BO_3_ units^33^. Bands at about 1097–1002 cm^-1^ are attributed to B–O stretching bond in the BO_4_ units^39^. Peaks at 989–924 cm^-1^ are mainly due to B–O stretching vibrations in BO_4_ groups^35^. Fine bands centered at around 898–822 cm^-1^ are assigned to B–O bond stretching in BO_4_ units^34^.

**Table 2:** Deconvolution parameters for IR spectra of the studied glass samples.

Sample	Peak positions and areas of the deconvoluted bands							
B-Na	C	1465.56	1456.25	1387.79	1344.64	1262.35	1097.39	1097.31
	A	74.55	12.41	3.80	6.57	51.41	23.30	55.26
B-Na-La	C	1447.60	1388.02	1059.63	935.74	709.66	496.51	470.58
	A	129.04	140.45	160.90	154.32	24.48	5.58	3.78
B-Na-Nd	C	1470.13	1413.08	1365.35	1318.42	1121.68	1008.65	1008.65
	A	86.77	3.59	7.64	36.71	82.71	1.27	56.85
B-Na-Gd	C	1495.72	1443.59	1401.38	1350.59	1287.78	1210.82	1084.65
	A	49.65	19.82	3.91	15.17	16.75	15.50	32.66
B-Na-Ho	C	1460.66	1414.99	1348.67	1236.11	1177.33	1069.77	985.93
	A	52.03	0.57	8.68	22.37	7.09	29.82	28.86
B-Na-Er	C	1447.38	1212.91	1102.12	964.29	844.19	707.96	441.15
	A	101.91	30.53	37.18	52.05	18.42	9.37	7.99
B-Na-Yb	C	1493.88	1424.04	1357.50	1263.58	1202.73	1133.48	1095.09
	A	32.66	11.61	27.70	5.78	3.46	19.21	35.75

Sample	Peak positions and areas of the deconvoluted bands							
B-Na	C	989.51	938.84	888.23	824.65	714.86	455.78	452.92
	A	88.15	5.80	46.78	29.64	55.62	32.98	4.16
B-Na-La	C	449.61	429.46	414.96	–	–	–	–
	A	7.10	5.87	1.72	–	–	–	–
B-Na-Nd	C	925.87	829.88	715.18	640.17	538.19	453.24	–
	A	104.30	12.11	28.73	12.39	1.69	15.00	–
B-Na-Gd	C	953.60	845.59	712.54	448.66	–	–	–
	A	44.36	15.40	11.93	3.77	–	–	–
B-Na-Ho	C	936.90	898.37	841.46	721.85	632.15	539.41	441.21
	A	9.14	9.92	24.51	17.79	8.90	5.42	21.13
B-Na-Er	C	–	–	–	–	–	–	–
	A	–	–	–	–	–	–	–
B-Na-Yb	C	1002.4	924.64	822.31	709.43	–	–	–
	A	40.69	24.87	29.58	5.58	–	–	–

**Fig. 5:**
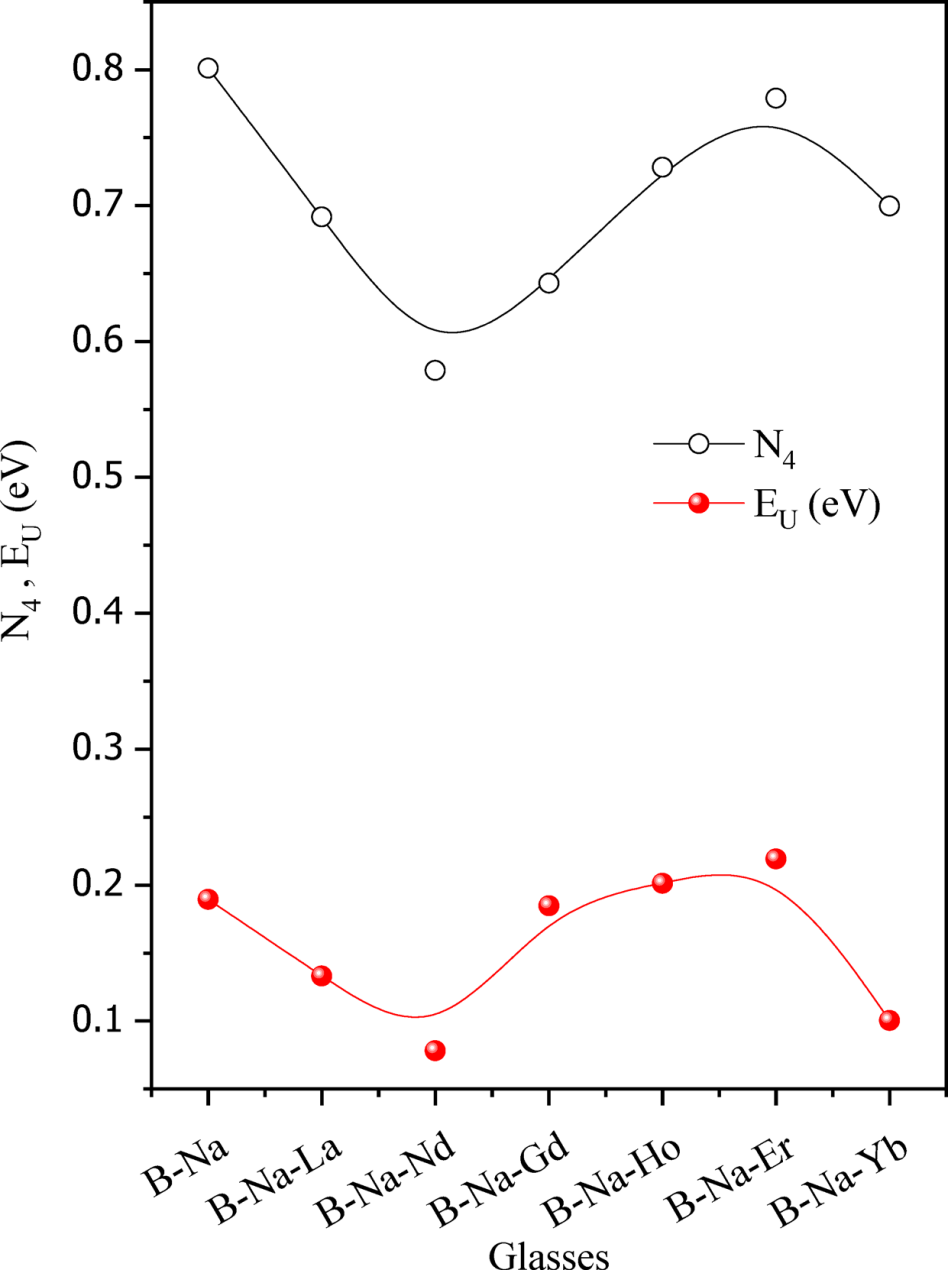
The N_4_ and E_U_ as a function of the B_2_O_3_-Na_2_O-RE_2_O_3_ glass samples.

To assess the impact of RE_2_O_3_ on the changes in the structural network, the fraction of four-coordinated boron atoms (N_4_) is calculated. The N_4_ borate units can be estimated as follows^34^:

1 $${{\varvec{N}}}_{4}=\frac{{{\varvec{A}}}_{4}}{{{\varvec{A}}}_{3}+{{\varvec{A}}}_{4}}$$

where A_4_ and A_3_ denote the areas corresponding to BO_4_ and BO_3_ units, respectively. Figure 5 displays the calculated N_4_ values for the glass samples under investigation. It is observed that N_4_ values are varying between 0.58 and 0.80. The N_4_ value is found to be the highest for the B-Na base glass (≈ 0.80), which reflects the majority spreading of the BO_4_ groups within the glass matrix. All the RE-doped glasses present a relatively lower value compared with the base glass. The lowest N_4_ value (≈ 0.58) is recorded for the B-Na-Nd glass, where the highest N_4_ value (≈ 0.78) for RE doped sample is recorded for the B-Na-Er glass. The observed fluctuation of the N_4_ values of the RE-doped glasses is suggested to be due to the differences in the field strength of these rare earth elements. RE ions with low field strength such as (La, Nd, and Gd) are reported to polarize the oxygen atoms in a manner weaker than that of the RE ions with high field strength such as Ho and Yb^40–47^. This behavior confirms the relatively high values of N_4_ for samples doped with Ho and Yb leading to the increase of BO_4_ units and the relatively low values of N_4_ for samples doped with La, Nd, and Gd leading to the decrease of BO_4_ units.

The structural modifications observed in the present RE-doped sodium borate glasses particularly the increase in BO₄ units and the disruption of boroxol rings are consistent with previous studies on modified borate systems. For example, Kolavekar et. al.^21^ found that TeO₂ incorporation into lead borate glasses doped with Pr₂O₃ improved thermal stability through structural compaction. Similarly, Hanamar et. al.^22^ reported that Sm₂O₃ addition to lithium zinc borate glasses enhanced BO₄ formation and led to greater photoluminescence efficiency and densification. K. A. Mahmoud et. al.^48^ showed that increasing Bi₂O₃ concentration in germanate–borate–tellurite glasses enhances network polymerization and optical transparency trends mirrored in our glasses with higher BO₄/BO₃ ratios. These effects parallel our findings, where rare earth doping promoted a denser borate network and altered optical response.

#### Density and molar volume calculations

**Fig. 6:**
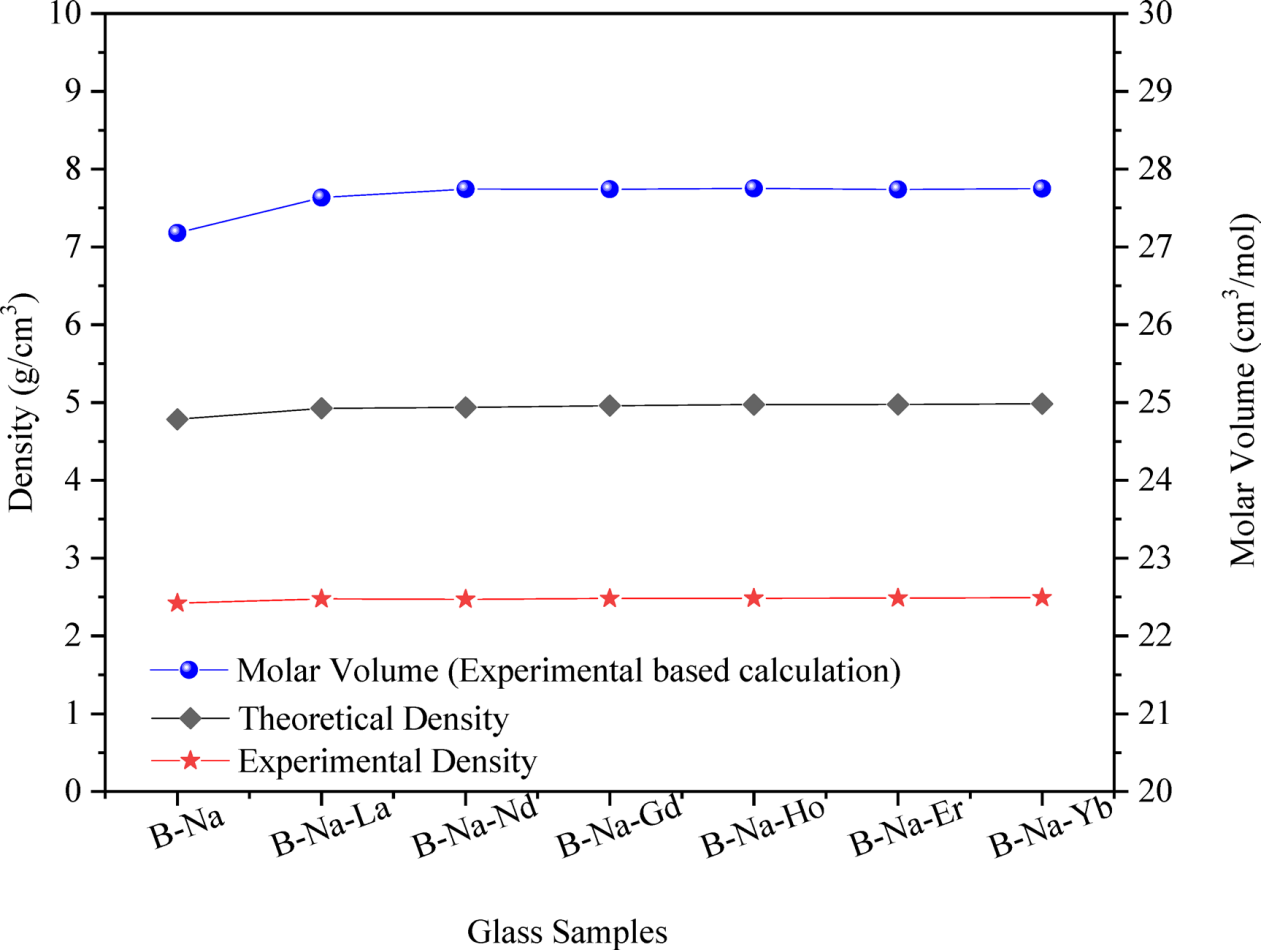
Experimental and theoretical densities, and calculated molar volume of the B_2_O_3_-Na_2_O-RE_2_O_3_ glasses.

The experimental density (${\rho }_{Ex}$) of the studied glasses is measured using the well-known Archimedes relation^33^:

2 $${{\varvec{\rho}}}_{{\varvec{E}}{\varvec{x}}}=\frac{{{\varvec{w}}}_{{\varvec{a}}}{{\varvec{\rho}}}_{{\varvec{t}}}}{{{\varvec{w}}}_{{\varvec{a}}}-{{\varvec{w}}}_{{\varvec{t}}}}$$

where, ${w}_{a}$, ${w}_{t}$, and ${\rho }_{t}$ are the sample weights in air, in toluene, and the density of toluene, respectively. As can be shown in Fig. 6, the value of ${\rho }_{Ex.}$ of all the RE-doped glasses has a higher magnitude when compared with the B-Na base glass. On the other hand, it can be noticed that the density value increases with a very low rate with replacing the RE elements in the direction from La to Yb. The continuous increase of the density value may be understood on the bases of the higher molar weights of the added RE oxide than that of the lower molar weight of the excluded Na_2_O. Similar behavior has been reported for various RE doped glass systems such as (Na_2_O)_35.7_ (RE_2_O_3_)_7.2_ (SiO_2_)_57.1_, (Na_2_O)_35.7_ (RE_2_O_3_)_7.2_ (GeO_2_)_57.1_, (Li_2_O)_35.7_ (RE_2_O_3_)_7.2_ (SiO_2_)_57.1_, and (K_2_O)_35.7_ (RE_2_O_3_)_7.2_ (SiO_2_)_57.1_ glasses, respectively^49–53^.

Additionally, the theoritical density (${\rho }_{Th}$) of the studied glasses is calculated using eq-3.^54^:

3 $${{\varvec{\rho}}}_{{\varvec{T}}{\varvec{h}}}=\frac{\sum {{\varvec{M}}}_{{\varvec{i}}}{{\varvec{x}}}_{{\varvec{i}}}}{\sum {{\varvec{V}}}_{{\varvec{i}}}{{\varvec{x}}}_{{\varvec{i}}}}$$

where ${M}_{i}$ is the molar weight, ${x}_{i}$ is the molar fraction, and ${V}_{i}$ is the packing factor parameter of each oxide. The packing factor values for the utilized oxides are extracted from Ref.^54^. As can be shown in Fig. 6, ${\rho }_{Th}$ values are found to represent a similar trend line to the ${\rho }_{Ex}$ values with comparatively higher values. This behavior may be due to several factors such as: The theoretical density is often calculated assuming a defect-free structure while real glass contains various types of imperfections such as voids, bubbles, and microcracks which decreases the actual density^55^. Also, the theoretical density calculation assumes an ideal composition based on the average formula. In practice, the composition can vary slightly due to manufacturing processes, leading to deviations in density^56^. Additionally, real glass can adsorb moisture from the environment or contain impurities that were not accounted for in the theoretical model^57^. These factors can decrease the actual density compared to the empirical value.

According to S. Inaba et. al.^54^ we can also determine the empirical density ${{\varvec{\rho}}}_{{\varvec{E}}{\varvec{m}}}$ from its chemical composition as follows:

4 $$\rho_{Em} = 0.5\frac{{\sum M_{i} x_{i} }}{{\sum V_{i} x_{i} }}$$

The molar volumes (${V}_{m}$) of the studied glasses are calculated by applying the expression:

5 $${V}_{m}=\frac{M}{{\rho }_{Ex}}$$

where *M* is the sample molecular weight. From Fig. 6, the values of the molar volume are found to slightly increase from 27.17 to 27.74 cm^3^/mol with the replacement of RE oxides on the expense of Na_2_O. It is worth mentioning that ${V}_{m}$ values show a similar behavior as the density values. This observation aligns with some previous studies that also found a proportional relationship between the density and molar volume of the examined glasses^58–60^. The continuous increase of the molar volume may be due to the larger ionic radii of the rare earth ions compared to sodium ions. Consequently, adding RE_2_O_3_ instead of Na_2_O makes the glass structure more open which correspondingly increases the ${V}_{m}$ of the glasses. Furthermore, the addition of RE oxides to the glassy structures also confirmed to form NBOs (see Sect. (3.1.2)), which may lead to weakening the glass structure and increasing molar volume^61^. Other studies have owned the proportionality between the density and molar volume to the decreasing of the bond strength, as well as to the increasing of the looseness of the glass matrix^62^.

The oxygen packing density (*OPD*) is also a key indicator of the compactness of the glass network. The *OPD* can be determined using the following^63^:

6 $$OPD=\frac{1000\times {N}_{O}}{{V}_{m}}$$

where ${N}_{O}$ is the number of oxygen atoms in the glass component. The *OPD* value is computed and presented in Table 3. The *OPD* is found to decrease by introducing RE_2_O_3_ to the base glass matrix. Decreasing of the *OPD* value may be because the rare earth ions disrupt the compactness and the packing of the glass network, leading to a less densely packed structure despite the increase in mass and volume^39,64^.

**Table 3:** Structural parameters of the studied glasses.

Physical property	Unit	Sample						
		B-Na	B-Na-La	B-Na-Nd	B-Na-Gd	B-Na-Ho	B-Na-Er	B-Na-Yb
Experimental density (*ρ*_*Ex*_)	g/cm^3^	2.421	2.477	2.471	2.480	2.485	2.488	2.491
Theoretical density (*ρ*_*Th*_)	g/cm^3^	4.785	4.925	4.937	4.958	4.971	4.975	4.984
Molar volume (*V*_*m*_)	cm^3^/mol	27.179	27.633	27.744	27.740	27.751	27.740	27.748
Oxygen Packing Density (*OPD*)	g.atom/l	73.586	73.101	72.808	72.818	72.789	72.820	72.797
Concentration of Rare Earth Ions (*N*_*RE*_)$\times {10}^{19}$	ions/cm^3^	–	4.58	4.42	4.12	3.96	3.91	3.81
Polaron Radius (*r*_*p*_)	Å	–	11.27	11.40	11.67	11.82	11.87	11.98
Interionic Distance (*r*_*i*_)	Å	–	27.96	28.28	28.96	29.34	29.45	29.73
Field Strength (*F*) )$\times {10}^{14}$	cm^-2^	–	2.36	2.31	2.20	2.15	2.13	2.09

To attain further insight into the compactness of the glass matrix, Eqs. 6, 7, 8 and 9^33^ are utilized to determine the concentration of rare earth ions (${N}_{RE}$) within the host matrix:

7 $${N}_{RE}=\frac{\left(mole\text{\% }R{E}_{2}{O}_{3}\right){N}_{A}}{{V}_{m}}$$

where ${N}_{A}$ is Avogadro’s number. The polaron radius (${r}_{p}$):

8 $${r}_{p}=\frac{1}{2}{\left(\frac{\pi }{6{N}_{RE}}\right)}^\frac{1}{3}$$

The distance between rare earth (RE) ions within the glass matrix can be approximated using the formula:

9 $${r}_{i}={\left(\frac{1}{{N}_{RE}}\right)}^\frac{1}{3}$$

Additionally, the field strength (*F*) corresponding to the RE ions is determined using the following Eq. ^33^:

10 $$F=\frac{Z}{{r}_{p}^{2}}$$

Where $Z$ is the valence of the RE ion. The calculated values of ${N}_{RE}$, ${r}_{p}$, ${r}_{i}$ and *F* are presented in Table 3. As the atomic weight of RE ions increases, their sizes and masses also increases. Firstly, the concentration of RE ions within the glass matrix $({N}_{RE})$ decreases as heavier ions tend to have larger ionic radii, making them less likely to fit into the host lattice of the glass. Secondly, the field strength *F* decreases as the atomic weight of the RE ions increases. This results in a greater shielding effect from inner electron shells. Consequently, the polaron radius increases due to reduced field strength, allowing a high mobility of charge carriers within the lattice. Finally, the interionic distance increases as the RE ions require more space within the lattice, leading to a decrease in the overall concentration of rare earth ions. These combined effects result in a nuanced interplay between the atomic weight of the rare earth dopant and various structural and electronic properties within the glass matrix.

### Optical spectroscopy

Ultraviolet–visible spectroscopy (UV–Vis) stands as a pivotal method for elucidating the electronic structure within glass networks, as well as for probing the optical transitions of materials and compounds^65^. The optical absorption coefficient (α) is computed using the formula^66^:

11 $$\alpha =\frac{2.303}{d}$$

**Fig. 7:**
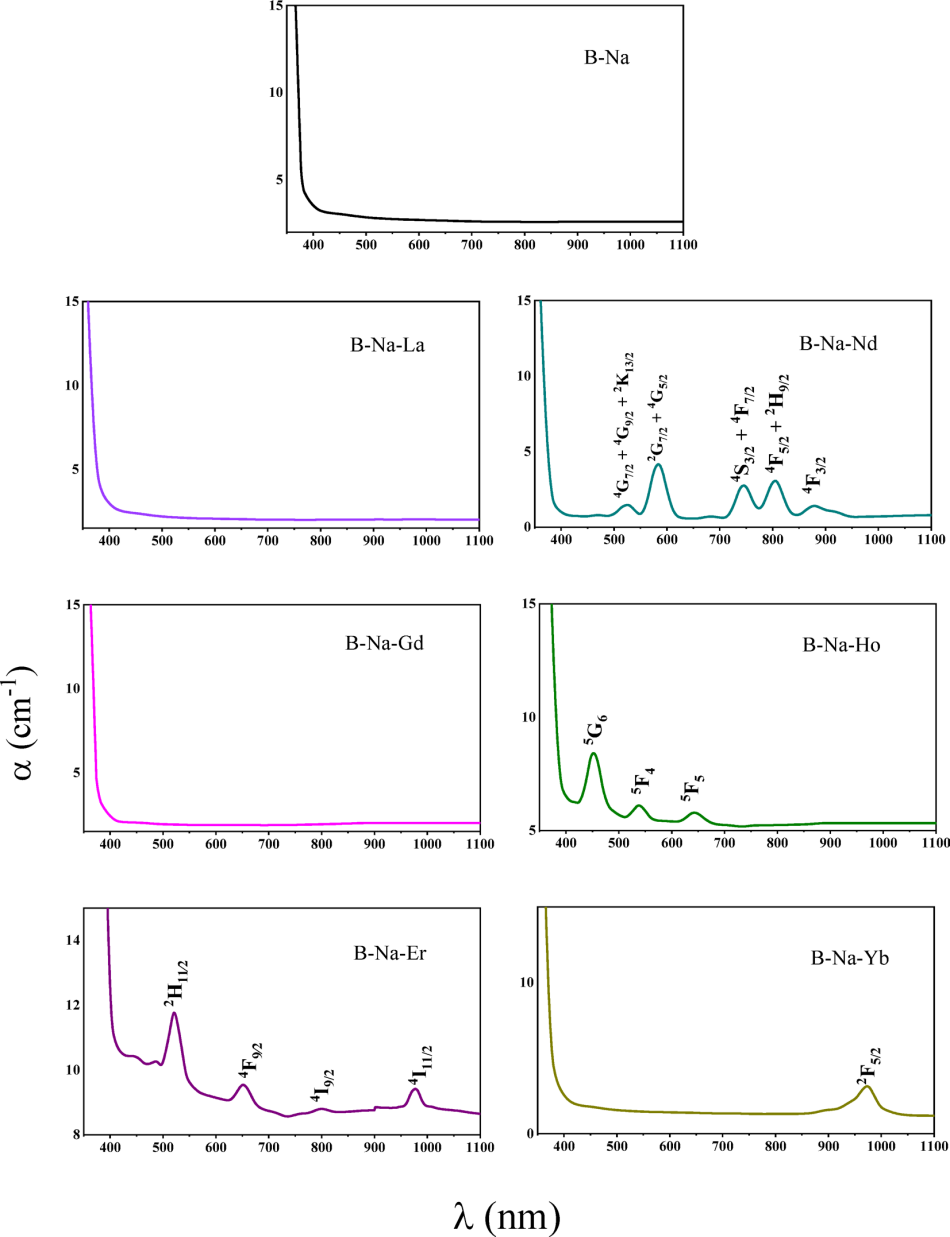
The absorption coefficient of the B_2_O_3_-Na_2_O-RE_2_O_3_ glasses as a function of wavelength.

Where, $d$ and *A* are the thickness and the absorbance of the studied glass, respectively. Figure 7 depicts the absorption coefficient of the investigated glasses as a function of wavelength in the range 200–1100 nm. All the glasses are characterized by a relatively high absorption coefficient from the start of measuring at 200 nm up to the absorption edge at about 400 nm. In the wavelength range of 400–1100 nm, all samples are characterized by a nearly constant value of α with some peaks that characterize the individual dopants (Nd^3+^, Ho^3+^, Er^3+^, and Yb^3+^ ) in the visible and FT-IR range of spectra. The absorption peaks are attributed to the transitions from the ground state to various excited states, as detailed in Table 4.

**Table 4:** Absorption peak wavelength of the B_2_O_3_-Na_2_O-RE_2_O_3_ glasses and their excitation levels.

Sample doped with	Peaks/nm	Excited state	Refs.
Nd_2_O_3_	524	^4^G_7/2_ + ^4^G_9/2_ + ^2^K_13/2_	^86^
	583	^2^G_7/2_ + ^4^G_5/2_	
	745	^4^S_3/2_ + ^4^F_7/2_	
	805	^4^F_5/2_ + ^2^H_9/2_	
	878	^4^F_3/2_	
Ho_2_O_3_	450	^5^G_6_	^87^
	537	^5^F_4_	
	640	^5^F_5_	
Er_2_O_3_	520	^2^H_11∕2_	^88^
	650	^4^F_9∕2_	
	800	^4^I_9/2_	
	977	^4^I_11/2_	
Yb_2_O_3_	972	^2^F_5/2_	^89^

#### Optical band gap energy (***E***_***g***_)

Two approaches are adopted to investigate the absorption phenomena near the absorption edge, as well as to elucidate the value of *E*_*g*_ and the type of electronic transition. Tauc’s approach^66^ is the first that used to calculate the *E*_*g*_ value according to the following relation:

12 $$\alpha \left(\nu \right)=\frac{B{\left(h\nu -{E}_{g}\right)}^{r}}{h\nu }$$

**Fig. 8:**
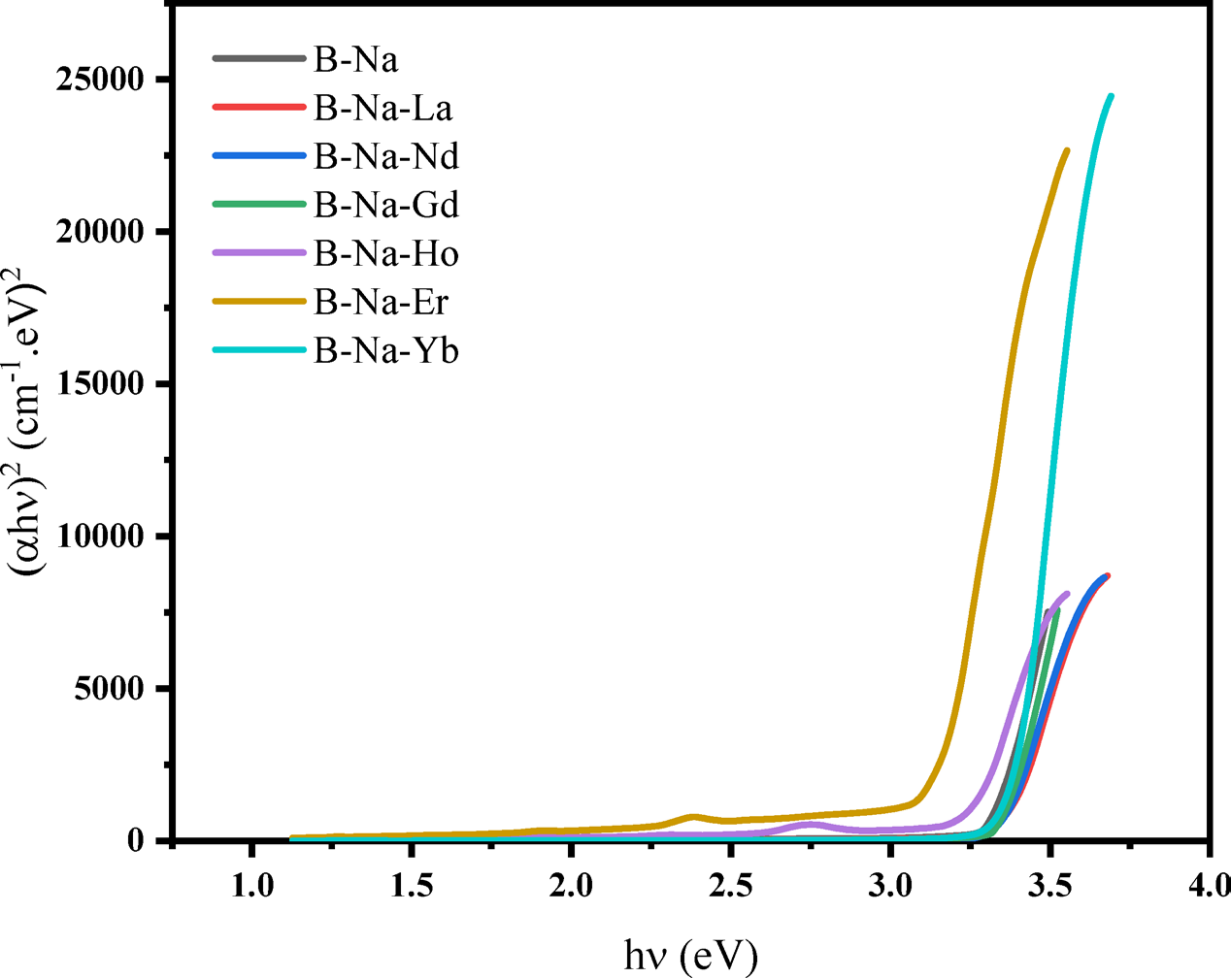
Tauc’s plots for direct allowed transitions (αhν)^2^ versus hν.

Where *B* is a constant, *hν* is the photon energy of the incident photon and *r* is an index for the type of transition with r = 1/2, 2, 3/2 or 3 corresponding to allowed direct, allowed indirect, forbidden direct, or forbidden indirect transitions, respectively^66^. According to the method by Zeyada et al.^67^ the value of r is found to be 0.54 at the absorption edge which highly recommends that the dominant transition is the allowed direct transition. Other authors^66,68,69^ also confirmed the possible allowed direct transitions to be dominant in different types of glasses. Tauc’s plot for direct allowed transition ((*αhν*)^2^ versus *hν)* for all glasses under investigation is presented in Fig. 8. The values of the direct ${E}_{g}$ transition are calculated and tabulated in Table 5. It’s worth mentioning that, adding 1 mol% of the La_2_O_3_ to the B-Na base glass is found to increase the value of ${E}_{g}$ from 3.314 to 3.365 eV. Changing the type of the rare earth added to the base glass with Nd_2_O_3_, Gd_2_O_3_, Ho_2_O_3_ and Er_2_O_3_, consequently, is found to continue decreasing the value of ${E}_{g}$ from 3.365 to 3.136 eV approximately, except for the sample B-Na-Yb, ${E}_{g}$ is found to increase again to ≈ 3.389 eV. Many authors^70–72^ had confirmed that the alternation from BO_4_ to BO_3_ groups in the borate based glasses increases the Non-Bridging Oxygen’s (NBOs), weakening the bonds, opening the glass network, and correspondingly increases the distances between the energetic states. This in turn may lead to the broadening of the energy gap and correspondingly the increase of the ${E}_{g}$ value. This is what exactly happened when the B-Na base glass is doped with La_2_O_3_ [La has an oxidation state of 3 + and has an electronic configuration of [Xe] 4f^0^ 5d^1^ 6s^2^] on the expense of the Na_2_O, which resulted in an increasing of the ${E}_{g}$ value. La^3^⁺ typically does not have low-lying f-electrons that could easily participate in electronic transitions. This results in minimal disruption to the glass electronic structure, maintaining a larger energy gap^73^. The decrease in ${E}_{g}$ value upon doping with Ho^3+^ and Er^3+^ signifies a broadening of both the valence and conduction bands, consequently leading to a narrow band gap^66^. The partially filled 4f. orbitals can interact more readily with the glass matrix, creating additional electronic states within the bandgap and facilitating electronic transitions and correspondingly decreasing the energy gap. Finally, the ${E}_{g}$ value of the B-Na-Yb sample is showed to increase again, and this may be due to the detected alternation from BO_4_ to BO_3_ groups, as well as, to the weak electronic transition between 4f. and 6 s states since the electronic configuration of Yb is [Xe] 4f^14^ 6s^2^. Yb^3^⁺ has nearly filled 4f. orbitals, making it stable and less interactive with the glass matrix, resulting in fewer mid-gap states and a larger energy gap value that is acceptable compared to the other rare earth metals^74–78^.

**Table 5:** The values of direct band gap energies ${E}_{g}$ from Tauc’s, ASF and HEM methods, parameters R, C_o_, Γ_c_, and Γ_1_ obtained from the fitting with Hydrogenic excitonic model (Eq. (14)), Urbach energy (E_U_), Steepness parameter (S) and refractive index (n).

Sample	${{\varvec{E}}}_{{\varvec{g}}}$(eV)			R(eV)	C_o_(eV^1/2^/Cm)	Γ_c_(eV)	Γ_1_(eV)	${{\varvec{E}}}_{{\varvec{U}}}$(eV)	*S*	${\varvec{n}}$
	Tauc	ASF	HEM							
B-Na	3.314	3.307	3.337	0.00148	398.65	0.03479	0.025	0.1894	0.1365	2.317
B-Na-La	3.365	3.374	3.360	0.00010	344.85	0.05097	0.03	0.1331	0.1942	2.305
B-Na-Nd	3.356	3.349	3.333	0.00010	334.18	0.02330	0.01	0.0780	0.3311	2.307
B-Na-Gd	3.340	3.339	3.353	0.00010	407.01	0.03304	0.02	0.1847	0.1399	2.310
B-Na-Ho	3.24	3.215	3.300	0.00500	327.55	0.06990	0.05	0.2012	0.1284	2.335
B-Na-Er	3.136	3.123	3.200	0.00495	467.66	0.07637	0.055	0.2192	0.1179	2.361
B-Na-Yb	3.389	3.379	3.369	0.00010	576.33	0.03213	0.02	0.1002	0.2578	2.299

**Fig. 9:**
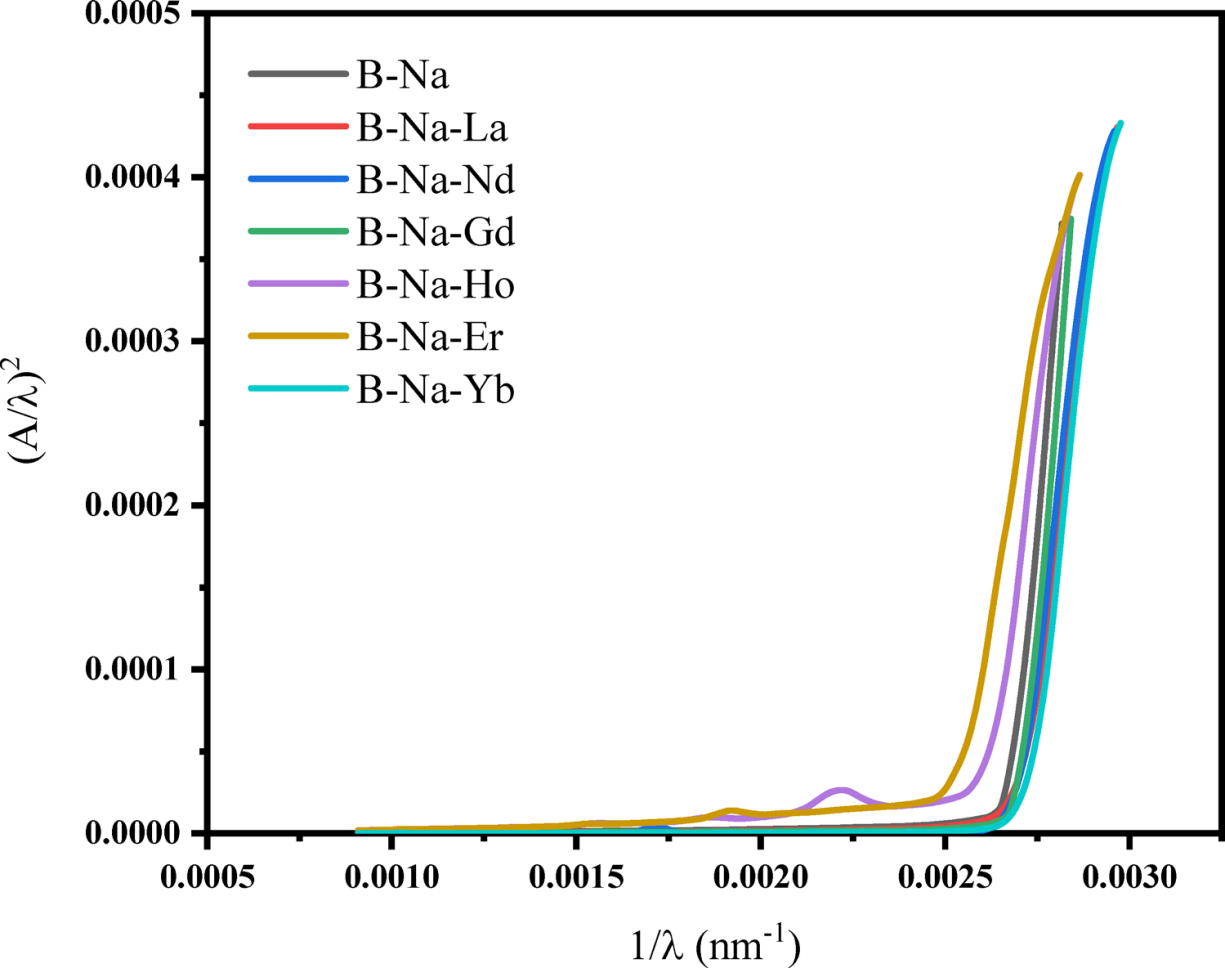
ASF plots for direct allowed transitions (A/λ)^2^ versus (1/λ) for glass samples.

The second approach is known as the absorption spectrum fitting (ASF). This approach developed by Souri and Shomalian^79^ along with Escobar-Alarcon et al. ^80^ and is used to assess the optical band gap. According the ASF model, the values of *α* can be expressed as a function of wavelength (*λ*) of the incident photon, and can be articulated as follows^66^:

13 $$\alpha \left(\lambda \right)=B{\left(hc\right)}^{r-1}\lambda {\left(\frac{1}{\lambda }-\frac{1}{{\lambda }_{g}}\right)}^{r}$$

where *h*, *c*, and ${\lambda }_{g}$ represent the Planck’s constant, the velocity of light, and the wavelength corresponding to the optical gap (absorption threshold), respectively. By employing Beer–Lambert’s law^66^, Eq. 12  can be reformulated as:

14 $${\rm A}\left(\lambda \right)=D\lambda {\left(\frac{1}{\lambda }-\frac{1}{{\lambda }_{g}}\right)}^{r}$$

where, $D = [B(hc{)}^{r-1}\times d/2.303]$. The optical band gap ${E}_{g}$ can be derived from the value of ${\lambda }_{g}$ using the relation ${E}_{g}=1239.83/{\lambda }_{g}$, where ${\lambda }_{g}$ is determined from extrapolating the linear region of $(A/\lambda {)}^{1/r}$ against $(1/\lambda )$​, where $(A/\lambda {)}^{1/r} = 0$. The graphical representation of $(A/\lambda {)}^{2}$ against $(1/\lambda )$ for direct allowed transition is depicted in Fig. 9. The ${E}_{g}$ values for direct allowed transition are presented in Table 5. The ${E}_{g}$ values obtained for allowed direct transition is found to be closely align with those obtained from Tauc’s plots.

Furthermore, Mott and Davis’s model^81–83^ overlooks the contributions of electron–hole pair (exciton) or electron-impurity interactions, which influence the optical transition rate by enhancing the formation of bound electron–hole pairs. Excitons are bound states of an electron and an electron hole attracted to each other by the Coulomb force^84–87^. There are many types of excitons, which are differ depending on the type of environment in which they are incubated, the type of molecules forming the network, and the bond strength between the molecules. The most important type of excitons to shed the light on here in this article is the Wannier-Mott excitons which characterizes the glassy materials^88^. They are found in materials with weakly bound electrons, such as semiconductors and glasses. These excitons have lower binding energies (few meV to tens of meV) and are more delocalized^88^. In glass, excitons are typically of the Wannier-Mott type due to the disordered structure of the material which allows for delocalized electron–hole pairs with relatively low binding energies^89^.

**Fig. 10:**
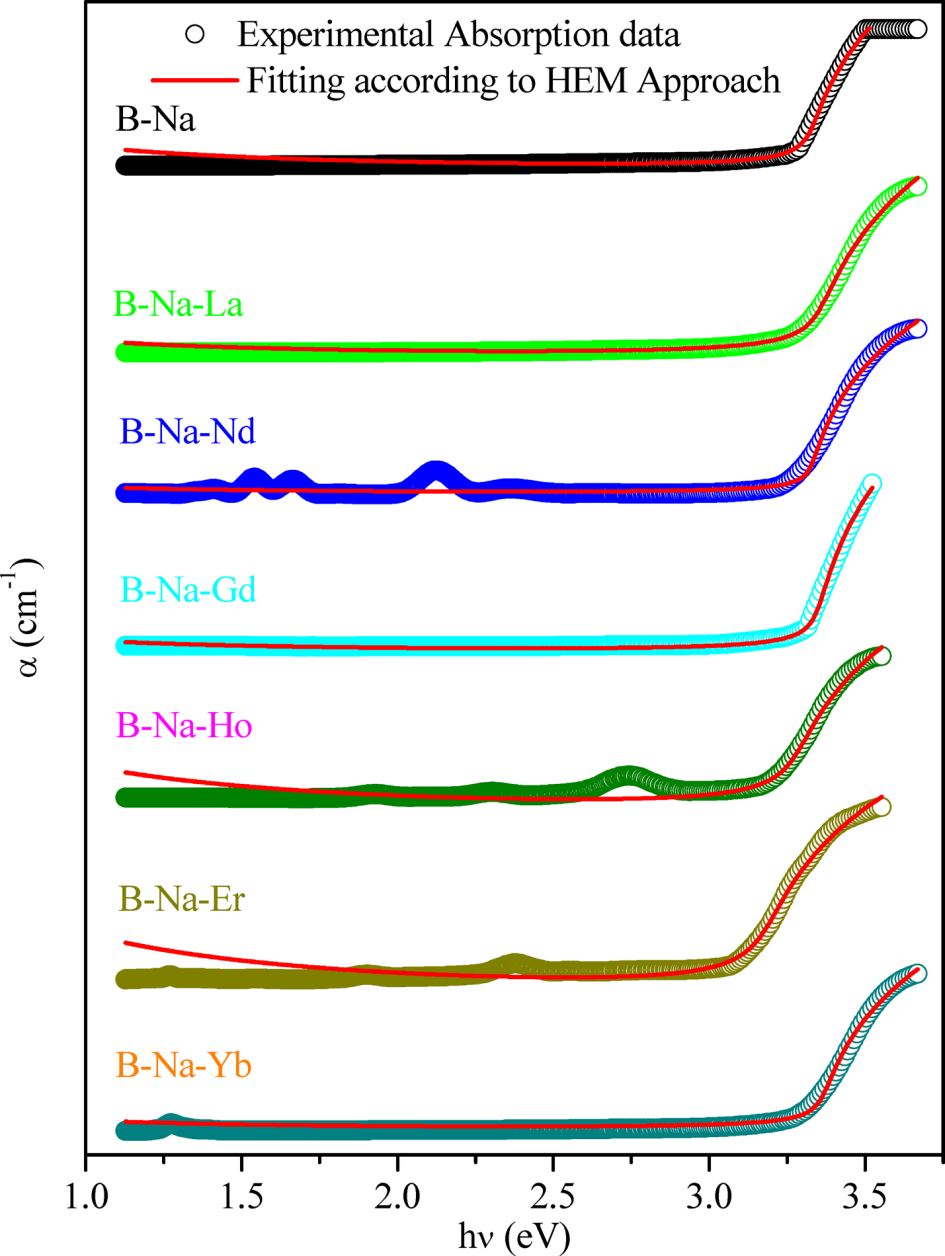
Fitting of abs orption coefficient (α) with Hydrogenic excitonic model (Eq. (14)) for the B_2_O_3_-Na_2_O-RE_2_O_3_ glass system.

To investigate the contribution of excitonic transitions in the studied glasses, the Hydrogenic Excitonic Model (HEM) proposed by Elliott^90–92^ is employed. Intrinsic parameters such as band gap and excitonic binding energy can be determined by fitting the experimental values of with the HEM approach as follows:

15 $$\alpha \left(E\right)=\frac{{C}_{0}{R}^{1/2}}{E}\left\{\sum_{m=1}^{\infty }\frac{2R}{{m}^{3}}\frac{{\Gamma }_{m}}{{\left(E-{E}_{m}\right)}^{2}+{\Gamma }_{m}^{2}}+\frac{1}{2}\left[\frac{\pi }{2}+arctan\left(\frac{\hslash \omega -{E}_{g}}{{\Gamma }_{c}}\right)\right]-\sum_{m=1}^{\infty }\frac{R}{{m}^{3}}\frac{{\Gamma }_{c}}{{\left(E-{E}_{m}\right)}^{2}+{\Gamma }_{c}^{2}}+\frac{\pi }{2}\frac{sinh(2{u}^{+})}{\mathit{cosh}\left(2{u}^{+}\right)-cos(2{u}^{-})}\right\}$$

where.

$${u}^{\pm }=\pi {\left(\frac{R}{2}\right)}^{1/2}{\left[\frac{{\left[{\left(E-{E}_{g}\right)}^{2}+{\Gamma }_{c}^{2}\right]}^\frac{1}{2}\pm \left(E-{E}_{g}\right)}{{\left(E-{E}_{m}\right)}^{2}+{\Gamma }_{c}^{2}}\right]}^{1/2}$$, $${E}_{m}={E}_{g}-\frac{R}{{m}^{2}}$$ and $${\Gamma }_{m}={\Gamma }_{c}-\frac{{\Gamma }_{c}-{\Gamma }_{1}}{{m}^{2}}$$ $$\text{with} m=1. 2. 3.\dots$$

where ${C}_{0}$ represents the absorption strength parameter, *R* denotes the excitonic binding energy, $E$ stands for the incident photon energy, ${E}_{g}$ signifies the optical band gap energy, and ${\Gamma }_{1}$ and ${\Gamma }_{c}$ denote the line widths of the $m = 1$ state and continuum, respectively. The initial term near the band edge generates a singular peak, centered at energy ${E}_{m}=1$, with an approximate width of ${\Gamma }_{1}$. The subsequent terms delineate the excitonic continuum, resulting in a step-like absorption edge of width ${\Gamma }_{c}$ above the band gap^93^. The experimental data fitted of α in comparison with the HEM approach is depicted in Fig. 10, considering only the line intensity of the free excitons ($m = 1$) and neglecting the higher values of exciton quantum number m^94^. The ${E}_{g}$ values obtained from the fitting by using the HEM model are added to Table 5 for comparison. Generally, in amorphous solids, both direct and indirect transitions are feasible owing to the absence of long-range order^90^. But, the obtained HEM based values of ${E}_{g}$ are found very close to those obtained by Tauc’s and ASF methods, which in turn strongly recommending that the dominant transition is of the allowed direct type. The other HEM outfitting parameters *R*, ${C}_{0}$,${\Gamma }_{c}$ , and ${\Gamma }_{1}$ are enumerated in. The goodness of the outfit parameter falls within the range of 0.995–0.998, indicating a commendable agreement between experimental and the theoretical data.

#### Urbach energy (E_U_)

**Fig. 11:**
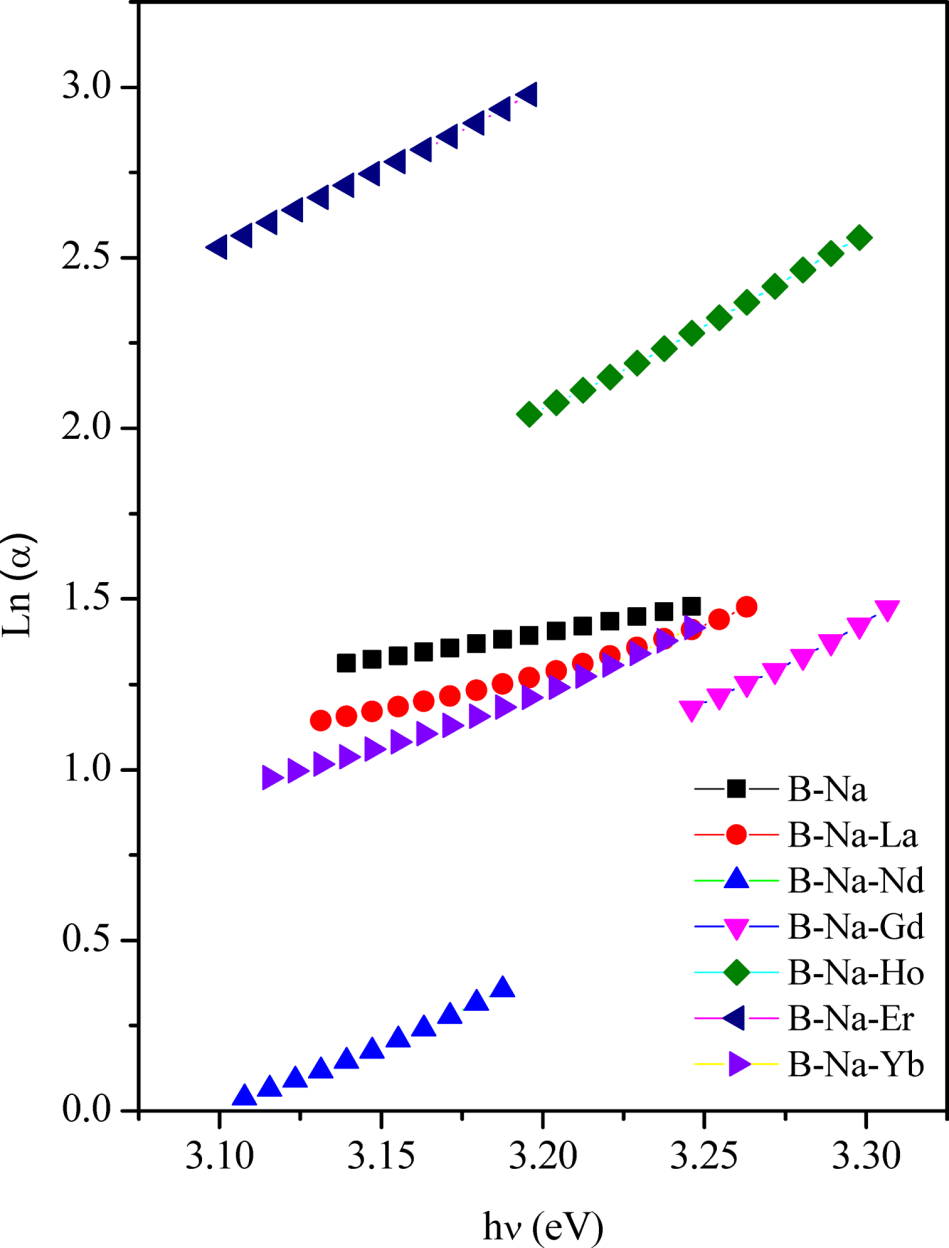
The relation of ln(α)versus photon energy (hν).

In amorphous materials, it is recognized that α exponentially depends on the incident photon energy near the absorption edge. This behavior can be explained according to the Urbach law^66^:

16 $$\alpha = \alpha _{o} e^{{hv/E_{U} }}$$

where ${\alpha }_{o}$ represents a pre-exponential constant, and ${E}_{U}$ denotes the Urbach energy corresponding to the width of the band tail of localized states. The magnitude of ${E}_{U}$ serves to assess the degree of disorderedly in amorphous materials. The broadening of the band tails associated with valence and conduction bands is attributed to the electron transitions between localized states, where the density of these localized states exponentially varies with energy^95^. Additionally, it reflects defect concentration. As a material exhibiting a large ${E}_{U}$_,_ this may indicate the formation of more defect states such as non-bridging oxygen (NBO) sites in glass^96^. Based on Eq. (15), a relation is plotted between $ln \alpha$ versus $h\nu$ and depicted in Fig. 11. The values of ${E}_{U}$ are determined and detailed in Table 5, as well as presented in Fig. 5 for comparison with the N_4_ values from the FT-IR analysis. It is observed from Fig. 5 that ${E}_{U}$ displays a similar trend as the variation of N_4_ values of the studied glasses with relatively small values. This strongly reflects the limited capacity of the formed defects within the glass network and recommends also that the Urbach energy is directly affected by the formation of BO_3_ and BO_4_ groups in the glass. The ${E}_{U}$ value is found to continue to decrease upon the introduction and change of the rare earth elements into the glass matrix until reaching the B-Na-Nd sample. This suggests that the glass network undergoes a rearrangement process which facilitated by the presence of rare earth ions, thereby reducing the number of defects, and consequently decreasing the E_U_ value^66^. On the other hand, in samples doped with Gd^3+^, Ho^3+^, and Er^3+^, the randomness is increased in the glass network thereby increasing the number of defects, and consequently increasing of ${E}_{U}$ value^66,95^. Finally, ${E}_{U}$ of the sample that doped with Yb^3+^ decreases again, indicating the formation of fewer defects, as well as a decrease in the localized states near the inter-band edge of the optical band gap^66,90,93,95^.

Furthermore, the electron–phonon or exciton-phonon interaction is established to contribute to the broadening of the absorption edge in glasses. This phenomenon gives rise to an optical parameter known as the steepness parameter (*S*), which is found oppositely depend on the ${E}_{U}$ values as follows^97^:

17 $$S=\frac{{k}_{\beta }T}{{E}_{U}}$$

where, *T* represents the temperature in Kelvin, and ${k}_{\beta }$ denotes the Boltzmann constant. The computed *S* parameter is presented in Table 5. It is observed that the *S* value is oppositely fluctuated with the ${E}_{U}$ value, which supports the low fluctuation of localized states within the band gap and the limited disorder range in the glass samples^97,98^.

#### Refractive index (n)

The refractive index (*n*) is an essential characteristic parameter that helps in finding a direct potential of the studied glasses. The magnitude of *n* is affected directly by the ${E}_{g}$ of the investigated glasses according to the following Eq. ^66^:

18 $$\frac{{n}^{2}-1}{{n}^{2}+2}=1-\sqrt{\frac{{E}_{g}}{20}}$$

The values of *n* for the studied glasses are calculated based on the ${E}_{g}$ values that estimated by Tauc’s method and presented in Table 5. Upon comparison with the B-Na base glass, the refractive index slightly decreases from 2.317 to 2.305 upon doping with La ion. The observed decrease in *n* can be elucidated by a reduction in the number of polarizable elements per unit volume. This decrease can also be linked to the reversion of some BOs to NBOs, thereby forming less covalent bonds^99^. Conversely, the *n* value is observed to continuously increase from 2.305 to 2.361 for samples doped with La, Nd, Gd, Ho and Er ions. This increase can be ascribed to the emergence of BOs that characterized by covalent bond properties. Consequently, they exhibit lower polarizability compared to NBOs, leading to enhanced cation refraction and a subsequent increase in the refractive index^66,100^. Finally, *n* is observed to drop down to 2.299 for the B-Na-Yb sample due to the increase of NBOs on the expense of the BOs and correspondingly the decrease of the polarizable elements density and n.

In this article, we shed light on the structural and optical properties of B-Na glass doped with various rare earth elements. And since these glasses have presented distinctive optical and structural properties, we are planning in the coming study to continue investigating the color and luminescence properties, as well as exploring the thermal and electrical conduction of these glasses.

M. I. Sayyed et. al.^101^ demonstrated that MgO incorporation into boro-germanate glasses improves density and refractive index via structural densification, similar to the density increases we observed in RE-modified glasses. M.H.A. Mhareb et. al.^102^ found that Nd^3^⁺ doping in B₂O₃–TeO₂–GeO₂–MgO glasses led to systematic improvements in optical clarity and radiation-shielding parameters, echoing our reported shifts in band gap. M. I. Sayyed et. al.^103^ reported that adding MoO₃ to borate-germanate-telluride glasses enhances both optical and ionizing radiation attenuation properties, supporting our conclusion that minor RE doping can meaningfully tune optical and structural properties for photonic and shielding purposes.

Furthermore, the optical band gap values in our RE-doped glasses (ranging from 3.13 to 3.38 eV) are in good agreement with those reported by Kolavekar et. al.^23^ for Bi₂O₃–Sm₂O₃ modified tellurite glasses, where similar shifts in band gap were attributed to increased non-bridging oxygen content and structural rearrangements. Hanamar et. al.^24^ also observed enhanced nonlinear optical properties in Sm^3^⁺-doped lithium zinc borate glasses, linked to compositional influences on the BO₃/BO₄ ratio. Compared to these studies, our work provides a broader comparative insight into how six different RE ions affect borate glass structure and optical transitions within a uniform host matrix.

## Conclusion

This study presents a comprehensive comparative analysis of sodium borate glasses doped with 1 mol% of various rare earth oxides (La₂O₃, Nd₂O₃, Gd₂O₃, Ho₂O₃, Er₂O₃, and Yb₂O₃), replacing an equivalent molar amount of Na₂O in a 50 mol% B₂O₃ – 50 mol% Na₂O base composition. XRD patterns confirmed the amorphous nature of all glasses.

FTIR spectroscopy revealed a progressive transformation of BO₃ to BO₄ units upon RE doping, accompanied by a decrease in boroxol ring intensity, indicating a denser and more polymerized borate network. The extent of structural reconfiguration varied systematically with the ionic radius and field strength of the dopant. These changes were reflected in the physical properties, where glass density increased from 2.42 to 2.49 g/cm^3^ and molar volume rose from 27.17 to 27.74 cm^3^/mol, highlighting the role of RE ions as compacting modifiers.

UV–Vis absorption analysis showed a decrease in absorption edge energy with increasing field strength of the dopant ion, and the optical band gap values ranged from 3.13 to 3.38 eV. Tauc, ASF, and HEM models consistently indicated allowed direct transitions in all compositions. The refractive index and metallization criterion values also reflected a systematic evolution with RE doping, correlating well with structural compactness and electronic polarizability.

Overall, this work provides a structure–property correlation framework for RE-doped sodium borate glasses. It demonstrates that minor substitutions of RE oxides induce quantifiable and predictable modifications in both short-range structure and optical response. These findings offer valuable guidelines for the design of borate-based glasses with tunable properties for applications in optoelectronics, photonics, and radiation shielding.

Although differential scanning calorimetry (DSC) and differential thermal analysis (DTA) could provide further insight into the thermal behavior and stability of the studied glasses, this aspect falls outside the scope of the current structural and optical investigation. These thermal studies are currently ongoing and will be presented in a future publication.

## Data Availability

The datasets generated during and/or analyzed during the current study are available from the corresponding author on reasonable request.
